# Characterization of peripheral cytokine-secreting cells responses in HIV/TB co-infection

**DOI:** 10.3389/fcimb.2023.1162420

**Published:** 2023-07-06

**Authors:** Yuting Tan, Wei Guo, Qi Zhu, Shihui Song, Yanni Xiang, Songjie Wu, Shi Zou, Yajun Yan, Ling Feng, Mingqi Luo, Ling Shen, Yong Feng, Ke Liang

**Affiliations:** ^1^ Department of Infectious Diseases, Zhongnan Hospital of Wuhan University, Wuhan, China; ^2^ Wuhan Research Center for Infectious Diseases and Cancer, Chinese Academy of Medical Sciences, Wuhan, China; ^3^ Department of Pathology, Zhongnan Hospital of Wuhan University, Wuhan, China; ^4^ Department of Pathology, School of Basic Medical Sciences, Wuhan University, Wuhan, China; ^5^ Wuhan Pulmonary Hospital, Wuhan Institute for Tuberculosis Control, Wuhan, China; ^6^ Department of Intensive Care Medicine, Yichang Central People’s Hospital, Yichang, Hubei, China; ^7^ Department of Nosocomial Infection Management, Zhongnan Hospital of Wuhan University, Wuhan, China; ^8^ Department of Microbiology and Immunology, Center for Primate Biomedical Research, University of Illinois College of Medicine, Chicago, IL, United States; ^9^ Department of Medical Microbiology, Wuhan University School of Basic Medical Sciences, Wuhan, China; ^10^ Hubei Engineering Center for Infectious Disease Prevention, Control and Treatment, Wuhan, China

**Keywords:** human immunodeficiency virus, tuberculosis, cytokine-secreting cells, TNF-α, anti-TB treatment

## Abstract

**Background:**

Currently the responses of peripheral cytokine-secreting cells in the natural course of human immunodeficiency virus (HIV) and tuberculosis (TB) co-infection haven’t been fully elucidated.

**Methods:**

The function of peripheral proinflammatory, regulatory and cytotoxic cytokine-secreting cells were investigated by direct intracellular cytokine staining (ICS) and flow cytometry, additionally, the absolute numbers of different cytokine-secreting cells were measured among patients with HIV/TB co-infection (HT group), and compared them with the healthy controls (HC group), patients with TB (TB group) and patients with HIV infection (HIV group). After one week’s anti-TB treatment, the changes of the percentages of cytokine-secreting cells were further evaluated in TB and HT groups.

**Results:**

Totally 26 individuals in the HC group, 51 in the TB group, 26 in the HIV group and 29 in the HT group were enrolled. The HT. HT group exhibited significantly lower absolute numbers of IFN-γ^+^CD4^+^, IFN-γ^+^CD8^+^, TNF-α^+^CD4^+^, IL17A^+^CD4^+^ T cells and TNF-α^+^CD14^+^ monocytes than the TB and HIV groups. Compared with the TB group, the percentages of CD8^+^ T cells secreting IFN-γ and perforin (p=0.010; p=0.043) were significantly lower among the HT group. Compared with the HIV group, the percentages of CD4^+^, CD8^+^ T cells and CD14^+^ monocytes secreting TNF-α (p=0.013; p=0.001; p<0.001) were significantly decreased, and the percentage of CD8^+^ T cells secreting IL-17A (p=0.015) was significantly increased among the HT group. Both the percentages of CD4^+^ T cells secreting TGF-β (p<0.001; p=0.001), and CD4^+^ and CD8^+^ T cells secreting granzyme A (all p<0.001), were significantly higher among the HT group than among the TB group and HIV group. After one week’s anti-TB treatment, an increased percentage of CD4^+^ T cells secreting TNF-α (p=0.003) was found in the TB group, and an increased percentage of CD8^+^ T cells secreting TNF-α (p=0.029) was found in the HT group.

**Conclusion:**

Significantly different functional profiles of peripheral proinflammatory, regulatory, and cytotoxic cytokine-secreting cells were observed in the natural course of HIV/TB co-infection compared to TB and HIV infection alone, even though the absolute numbers of those cells were significantly lower in HIV/TB co-infection. TNF-α-secreting CD8^+^ T cells may be a more sensitive marker for early evaluation of anti-TB treatment efficacy in patients with HIV/TB co-infection.

## Introduction

Tuberculosis (TB), caused by *Mycobacterium tuberculosis* (MTB) infection, is the most common opportunistic infection in human immunodeficiency virus (HIV)-infected patients. MTB is an intracellular bacterium that dominantly inducing cellular immune responses, including pro-inflammatory cytokine secretion like IL-1, IL-6 and IFN-γ, cytotoxic cytokine release like granzyme and perforin, and regulatory cytokine production like IL-10 and TGF-β (Abebe et al., 2006). Besides, innate immune responses, like TNF-α secretion by monocytes, also act at the initial stage of MTB infection to recruit inflammatory cells to the infected location and induce adaptive immune responses (Nunes-Alves et al., 2014). In HIV infection, CD4^+^ T cells are progressively destroyed, and the function of other cell subpopulations, such as Th17 and Th22 cells, are also depressed gradually (Bell and Noursadeghi, 2018), while chronic MTB infection may also cause exhaustion of T cells’ function (Wong et al., 2018). Moreover, in both MTB infection and HIV infection, dysregulated T-cell function may potentially be associated with altered immune cell metabolism (Wik and Skålhegg, 2022). All those suggest that MTB infection and HIV infection may share some common features, and TB with or without HIV infection may have different immune responses. Previous studies found that MTB-specific T cells were prior to depletion in early HIV infection in comparison to the total memory T cell populations or cytomegalovirus-specific memory T cells, supporting that the depletion of T cell populations caused by HIV contributes to the increased risk of TB (Bell and Noursadeghi, 2018). Though HIV/TB co-infection influence each other to exacerbate disease progression, little is known on how human immune system reacts to HIV/TB co-infection and how anti-TB treatment affects the immune responses.

Previous studies have investigated the cytokine responses during MTB infection to inspect the initial immune status (Abebe et al., 2006; Nemeth et al., 2012; Rozot et al., 2013), or monitor the changes of cellular responses before and after anti-TB treatment (Saharia et al., 2016). Some studies also described the immune status in HIV/TB co-infection (Chiacchio et al., 2014; Brighenti and Joosten, 2018). In these studies, different experimental methods were performed and the most common techniques were enzyme linked immunosorbent assay (ELISA), flow cytometry of the cytokine-secreting cells with specific antigen stimulation. However, ELISA can’t provide the source of each cytokine, and flow cytometry with specific MTB antigen stimulation may reflect the cells’ response to MTB but can’t represent the actual immune state in human since there is seldom such a high concentration of antigens in blood. Up till now, the panorama of cellular responses in the natural course of HIV/TB co-infection hasn’t been fully elucidated.

Direct intracellular cytokine staining (ICS), a dependable method that without specific antigen stimulation during the experiment, has been well used in our previous studies to access the natural cellular responses in TB and/or HIV infection (Tan et al., 2022; Zou et al., 2022a; Zou et al., 2022b). In the present study, direct ICS assay was performed to profile the function of proinflammatory, regulatory and cytotoxic cytokine-secreting cells in HIV/TB co-infection, and compared them with the healthy controls, mono-TB and mono-HIV infection. And the changes of those cytokine-secretion cells after short-term anti-TB treatment were also evaluated. Our study found that despite the obviously decreased absolute numbers of peripheral pro-inflammatory, regulatory and cytotoxic cytokine-secreting cells in HIV/TB co-infection, different functional profile of these cells existed in the natural course of HIV/TB co-infection when compared with TB and HIV infection alone. TNF-α-secreting CD8^+^ T cells might be more sensitive for evaluating the early efficacy of anti-TB treatment in patients with HIV/TB co-infection.

## Methods

### Study population

Subjects enrolled between May, 2018 and December, 2020 were divided into four groups: 1) Healthy controls (HC) group: individuals without any infection within 2 weeks before enrollment were recruited from the physical examination center in Zhongnan Hospital of Wuhan University. HIV infection and MTB infection were excluded by HIV antibody screening, chest X-ray and interferon gamma release assay (IGRA). 2) TB group: patients with confirmed TB before anti-TB treatment (verified by smear or culture positive, and/or MTB DNA test positive, and/or Xpert MTB/RIF test positive, and/or histopathological evidence) were recruited from Wuhan Pulmonary Hospital. All had no HIV infection before enrollment. 3) HIV group: patients with confirmed HIV infection were recruited from AIDS Clinical Guidance and Training Center, Zhongnan Hospital of Wuhan University. All received no antiretroviral therapy (ART) and had no MTB infection before enrollment; 4) HIV/TB co-infection (HT) group: HIV-infected patients with confirmed TB before anti-TB treatment were recruited from department of infectious diseases, Zhongnan Hospital of Wuhan University. All received no ART before enrollment. TB group and HT group enrolled were also followed up at one week after anti-TB treatment.

### Monocytes, lymphocyte, CD4 and CD8 counts measurement

Routine blood tests were performed in all participants for obtaining the monocyte counts and lymphocyte counts. CD4 and CD8 counts of all participants were measured by flow cytometry with the whole peripheral blood using human CD45^+^, CD3^+^, CD4^+^ and CD8^+^ cell markers (BD Biosciences) and BD Trucount™ tubes according to the manufacturer’s instructions.

### Samples collection and isolation of PBMCs

Peripheral blood were collected from all participants at enrollment. For TB group and HT group, peripheral blood were also collected at one week after anti-TB treatment. Peripheral blood mononuclear cells (PBMCs) were isolated from freshly collected EDTA coagulated blood by Lymphoprep (Axis-Shield, Norway) with density gradient centrifugation. Cell pellets were treated with 5 ml RBC lysis buffer (Sigma-Aldrich) for 10 min, followed by washing once with 5% FBS-PBS. PBMCs were then counted and cryopreserved at -80°C until next step experiments.

### Antibodies and reagents

These following antibodies (Abs) were used for surface marker staining and ICS combined with flow cytometry (all Abs were from Biolegend): anti-CD3-PerCP-cy5.5 (UCHT1), anti-CD3-FITC (UCHT1), anti-CD3-PE (HIT3a), anti-CD4-APC (OKT4), anti-CD4-APC-cy7 (RPA-T4), anti-CD8-PE (RPA-T8), anti-CD8-APC-cy7 (SK1), anti-CD14-FITC (HCD14), anti-IFN-γ-PE (4S.B3), anti-TNF-α-APC (MAb11), anti-IL-17A-PE-cy7 (BL168), anti-Foxp3-PE (206D), anti-TGF-β-PE-cy7 (TW4-2F8), anti-Perforin-PC (dG9), anti-granzyme A-PE (CB9),.

### Direct intracellular cytokine staining

This procedure was performed as described before (Tan et al., 2022; Zou et al., 2022a; Zou et al., 2022b). Direct ICS was adapted from the original ICS protocol that PBMCs were directly measured for intracellular cytokines detection without prior *in vitro* specific antigen stimulation. PBMCs were incubated for 1 h with medium in the presence of anti-CD28/CD49d (1 mg/ml, Biolegend) in a 200 μL final volume in round-bottom 96-well plates at 37°C, 5% CO_2_, followed by a 5 hours’ incubation in the presence of brefeldin A (GolgiPlug; BD Biosciences). At the end of the incubation, cells were washed once with 2% FBS-PBS and stained at room temperature for at least 15-30 min with surface marker Abs (CD3, CD4, CD8 and CD14). After the next 45 min permeabilization (Cytofix/Cytoperm; BD Biosciences), another 45 min for the above intracellular cytokine staining was performed. Finally, cells were re-suspended in 2% formaldehyde and subjected to flow cytometry analysis. The percentages of different cytokines-secreting cells among the four groups were accessed, and the absolute numbers (/ul) of different cytokines-secreting cells were obtained according to the percentages and counts of CD4^+^, CD8^+^ T cells and monocytes.

### Statistical analysis

Flow cytometric data were analyzed with FlowJo version 7.6.1 for Windows. SPSS 21.0 and Graphpad Prism 5.0 were used for data statistics and plotting. Variables are denoted as the median (IQR) or n (%). The comparisons of count data between groups used Chi-square test. For single comparisons, we performed a Mann Withney U test. For multiple comparisons, Kruskall-Wallis test followed by Dunn-Bonferroni *post-hoc* test was used. Comparison of two matched results from one individual donor before and after anti-TB treatment used the paired rank sum test.

## Results

### Characteristics of study participants

Totally 132 individuals were enrolled in this study. Of them, 26 were in the HC group, 51 were in the TB group, 26 were in the HIV group and 29 were in the HT group. Characteristics of participants in each group were shown in **Table 1**. The proportions of males in the TB, HIV and HT group were higher than that in the HC group. There was no significant difference in the proportion of comorbidities among the four groups, and the proportion of pulmonary TB was not significantly different between the TB and HT group. The lymphocyte counts, CD4^+^ T lymphocyte counts (CD4 counts) and CD8^+^ T lymphocyte counts (CD8 counts) among the HT group were lower than that among the HC, TB and HIV group.

**Table 1:** Characteristics of participants enrolled in the study.

	HC (n=26)	TB (n=51)	HIV (n=26)	HT (n=29)	p value
age [years, median (IQR)]	32 (25-59)	36 (31-63)	31 (29-54)	35 (28-58)	0.240
Male, n (%)	7 (26.9)	33 (64.7)	24 (92.3)	24 (82.8)	<0.001
Comorbidities^§^, n (%)	2 (7.7)	6 (11.7)	4 (15.3)	5 (17.2)	0.715
Pulmonary TB, n (%)	/	30 (58.8)	/	14 (48.3)	0.362
HIV-VL [copies/ml, median (IQR)]	/	/	33767 (22503-77958)	55615 (34123-98690)	0.108
Lymphocyte count [/µl, median (IQR)]	1624 (1411-1817)	1133 (914-1422)	1000 (802-1306)	477 (223-673)	<0.001
CD4 count [/µl, median (IQR)]	952 (793-1102)	677 (555-969)	422 (266-602)	92 (39-145)	<0.001
CD8 count [/µl, median (IQR)]	648 (516-852)	409 (254-500)	620 (500-905)	389 (331-482)	0.041

^§^Refers to have one of the following diseases: diabetes, cardiovascular and cerebrovascular diseases, chronic lung diseases, chronic kidney diseases, chronic liver diseases.

### Absolute numbers of different cytokine-secreting cells in HT group

**Figure 1:**
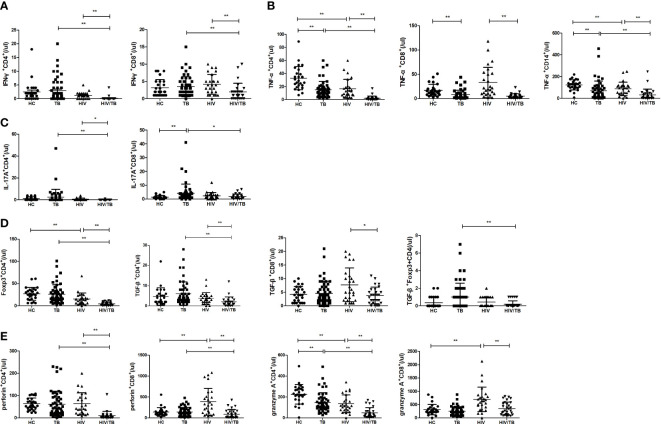
Comparisons of absolute numbers (/ul) of different cytokine-secreting cells between the HC, TB, HIV and HT group. **(A)** absolute numbers of IFN-γ-secreting CD4^+^ and CD8^+^ T cells; **(B)** absolute numbers of TNF-a-secreting CD4^+^ and CD8^+^ T cells, and CD14^+^ monocytes; **(C)** absolute numbers of IL-17A-secreting CD4^+^ and CD8^+^ T cells; **(D)** absolute numbers of Foxp3-secreting CD4^+^ T cells, TGF-β-secreting CD4^+^ and CD8^+^ T cells, and TGF-β-secreting Foxp3^+^CD4^+^ T cells; **(E)** absolute numbers of perforin-secreting CD4^+^ and CD8^+^ T cells; **(F)** absolute numbers of granzyme A-secreting CD4^+^ and CD8^+^ T cells. *, p<0.05 and **, p<0.01. HC: health controls; TB: tuberculosis; HIV: HIV infection; HT: HIV/TB co-infection.

We first analyzed the absolute numbers of different proinflammatory, regulatory and cytotoxic cytokine-secreting cells in the HT group, and compared with the TB and HIV group. As shown in **Figure 1**, the absolute numbers of IFN-γ^+^CD4^+^, IFN-γ^+^CD8^+^, TNF-α^+^CD4^+^, IL17A^+^CD4^+^ T cells and TNF-α^+^CD14^+^ monocytes in the HT group were significantly lower than that in the TB (p<0.001, p=0.001, p<0.001, p=0.001 and p=0.007) and HIV group (p<0.001, p=0.001, p<0.001, p=0.039 and p<0.001), which might be associated with the significantly lower CD4 and CD8 counts caused by HIV/TB co-infection.

### Function of different cells to secrete pro-inflammatory cytokine in HT group

**Figure 2:**
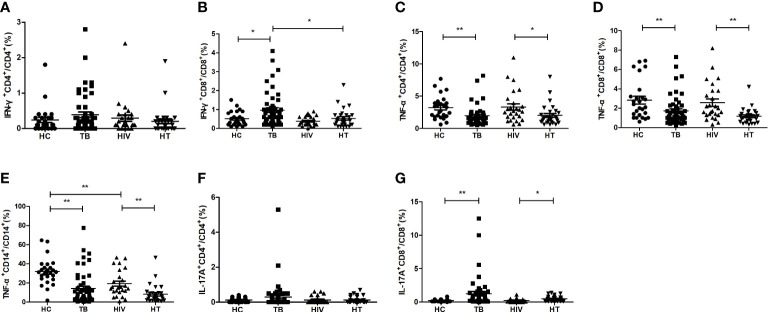
Comparisons of percentages of proinflammatory cytokine-secreting cells between the HC, TB, HIV and HT group. **(A)** percentage of IFN-γ-secreting CD4^+^ T cells in CD4^+^ T cells; **(B)** percentage of IFN-γ-secreting CD8^+^ T cells in CD8^+^ T cells; **(C)** percentage of TNF-a-secreting CD4^+^ T cells in CD4^+^ T cells; **(D)** percentage of TNF-a-secreting CD8^+^ T cells in CD8^+^ T cells; **(E)** percentage of TNF-a-secreting CD14^+^ monocytes in CD14^+^ monocytes; **(F)** percentage of IL-17A-secreting CD4^+^ T cells in CD4^+^ T cells; **(G)** percentage of IL-17A-secreting CD8^+^ T cells in CD8^+^ T cells. *, p<0.05 and **, p<0.01. HC: health controls; TB: tuberculosis; HIV: HIV infection; HT: HIV/TB co-infection.

Further, the percentages of different proinflammatory cytokine-secreting cells were analyzed between the four groups (shown in **Figure 2**). Compared with the TB group, the percentage of CD8^+^ T cells secreting IFN-γ was significantly lower in the HT group (p=0.010). The percentage of CD8^+^ T cells secreting IL-17A was significantly increased (p=0.015) and the percentages of CD4^+^ and CD8^+^ T cells secreting TNF-α, and percentage of CD14^+^ monocytes secreting TNF-α were significantly decreased (p=0.013; p=0.001; p<0.001) in the HT group than in the HIV group. Compared to the HC group, the TB group showed a significant increase in the percentages of CD8^+^ T cells secreting IFN-γ and IL-17A (p=0.020 and p<0.001, respectively), and a significant decrease in the percentages of CD4^+^, CD8^+^ T cells and CD14^+^ monocytes secreting TNF-α (p<0.001, p=0.004, and p<0.001, respectively). The percentage of CD14^+^ monocytes secreting TNF-α was significantly decreased in the HIV group than in the HC group (p=0.002). Those findings suggest that chronic TB may inhibit the expression of TNF-α or induce TNF-α-secreting cells depletion, or TNF-α-secreting cells may migrate to the lesion site.

### Function of different cells to secrete regulatory cytokine in HT group

**Figure 3:**
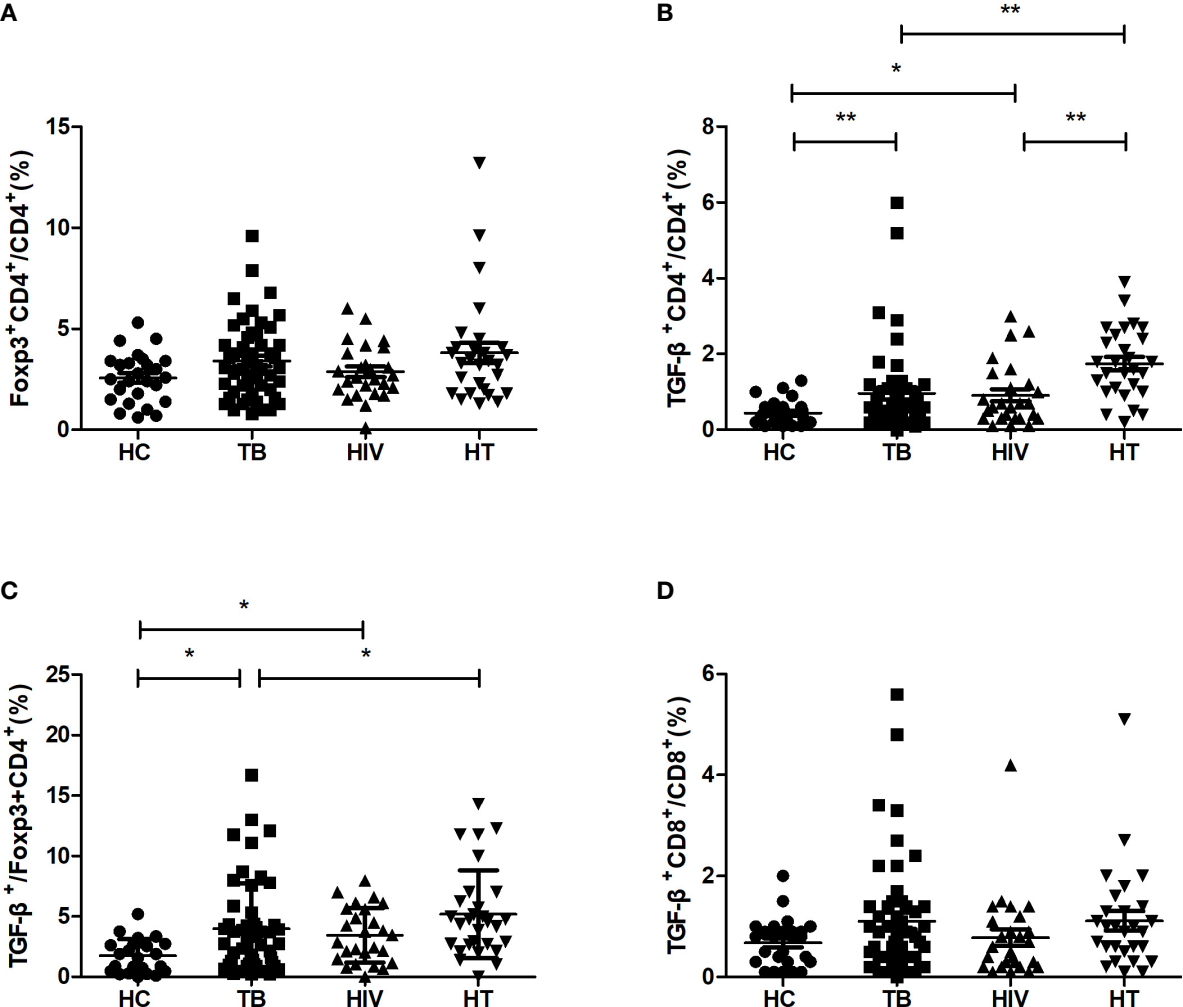
Comparisons of percentages of regulatory cytokine-secreting cells in the HC, TB, HIV and HT group. **(A)** percentage of Foxp3-secreting CD4^+^ T cells in CD4^+^ T cells; **(B)** percentage of TGF-β-secreting CD4^+^ T cells in CD4^+^ T cells; **(C)** percentage of TGF-β-secreting Foxp3^+^CD4^+^ T cells in Foxp3^+^CD4^+^ T cells; **(D)** percentage of TGF-β-secreting CD8^+^ T cells in CD8^+^ T cells. *, p<0.05 and **, p<0.01. HC: health controls; TB: tuberculosis; HIV: HIV infection; HT: HIV/TB co-infection.

The percentages of regulatory cytokine-secreting cells were compared between the four groups. As shown in **Figure 3**, there were no significant differences of the percentages of CD4^+^ T cells expressing Foxp3 between the HT, HIV, TB and HC groups. The percentage of CD4^+^ T cells secreting TGF-β in the HT group was significantly higher than that in both TB and HIV group (p<0.001; p=0.001). The percentages of Foxp3^+^CD4^+^ T cells secreting TGF-β were significantly higher in both TB group and HIV group than in HC group (p=0.029; p=0.026), and the HT group had a higher percentage of Foxp3^+^CD4^+^ T cells secreting TGF-β than the TB group (p=0.038), suggesting that HIV/TB co-infection may enhance the ability of CD4^+^ Treg cells to secrete TGF-β.

### Function of different cells to secrete cytotoxic cytokine in HT group

**Figure 4:**
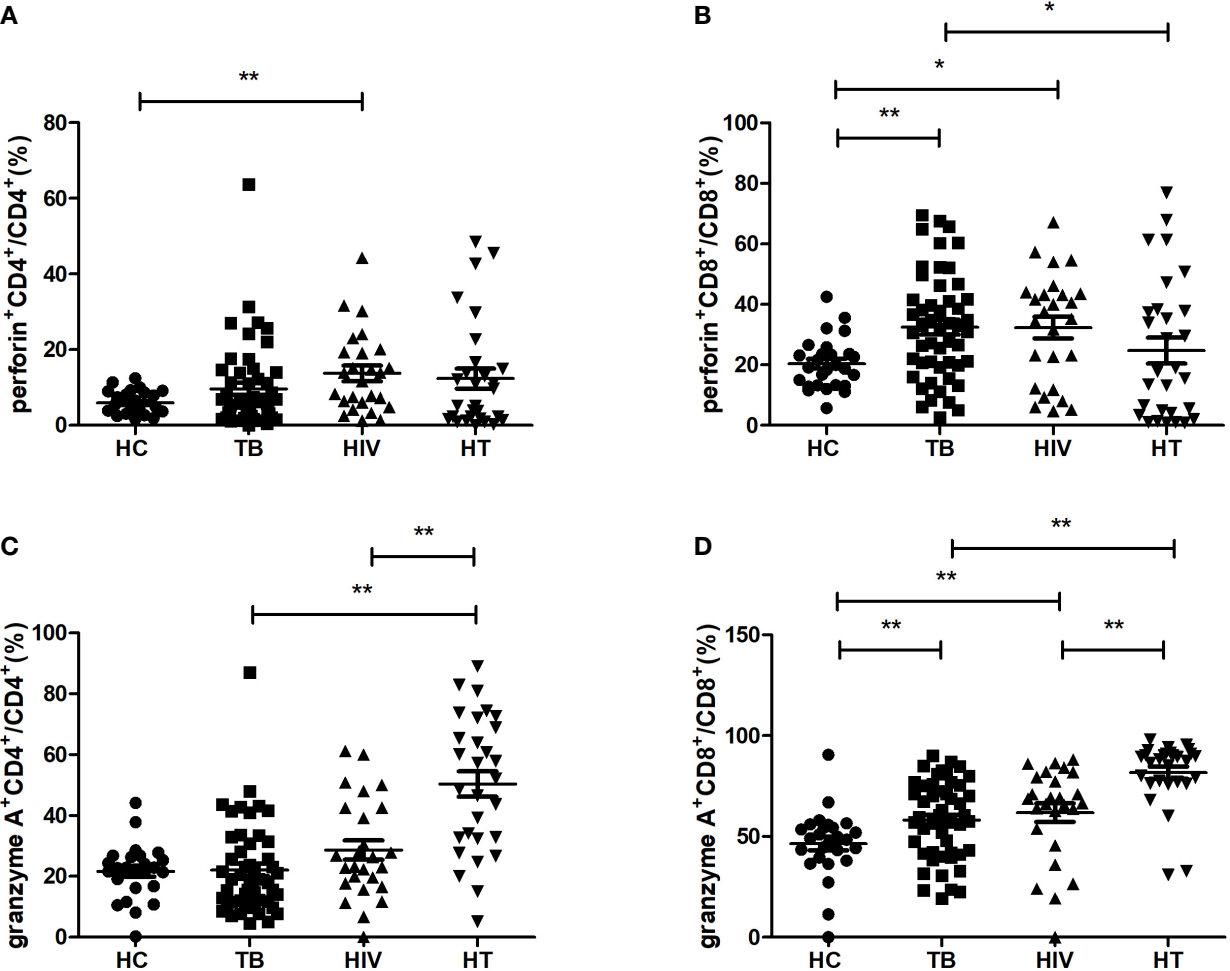
Comparisons of percentages of cytotoxic cytokine (granzyme A, perforin)-secreting cells in the HC, TB, HIV and HT group. **(A)** percentage of perforin-secreting CD4^+^ T cells in CD4^+^ T cells; **(B)** percentage of perforin-secreting CD8^+^ T cells in CD8^+^ T cells; **(C)** percentage of granzyme A-secreting CD4^+^ T cells in CD4^+^ T cells; **(D)** percentage of granzyme A-secreting CD8^+^ T cells in CD8^+^ T cells. *, p<0.05 and **, p<0.01. HC, health controls; TB, tuberculosis; HIV, HIV infection; HT, HIV/TB co-infection.

For realizing the function of different cytotoxic cytokine-secreting cell subsets among HIV/TB co-infection, the percentages of cytotoxic cytokine-secreting cells were compared between the four groups. As shown in **Figure 4**, in comparison to the HC group, the TB group had significantly higher percentages of CD8^+^ T cells secreting perforin and granzyme A (p=0.002; p=0.008), while the HIV group had significantly higher percentages of CD4^+^ T cells secreting perforin, CD8^+^ T cells secreting perforin and granzyme A (p=0.004; p=0.018; p=0.002). The HT group had significantly higher percentages of CD4^+^ and CD8^+^ T cells secreting granzyme A, respectively, compared to both TB and HIV groups (all p<0.001). These findings suggest that the expansion of granzyme A-secreting CD4^+^ and CD8^+^ T cells may play a critical role in viral and MTB clearance in HIV/TB co-infection.

Moreover, we also found that the percentage of CD8^+^T cells secreting perforin in the HT group was lower than that in the TB group (p=0.043), which indicates that there may be a differential regulation of perforin and granzyme A production in CD8^+^ T cells in the HT group when compared with the TB group.

### Significant change of TNF-α-secreting cells after short-term anti-TB treatment

**Figure 5:**
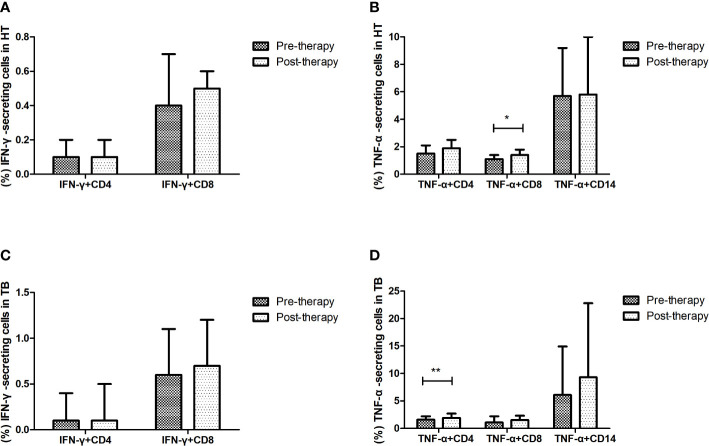
Effect of short-term anti-TB treatment on IFN-γ-secreting and TNF-a-secreting cells in HT and the TB group. **(A)** Changes of the percentages of IFN-γ-secreting CD4^+^ T cells and CD8^+^ T cells pre-therapy and post-therapy in the HT group; **(B)** Changes of the percentages of TNF-a-secreting CD4^+^ T cells and CD8^+^ T cells, and TNF-a-secreting CD14^+^ monocytes pre-therapy and post-therapy in the HT group; **(C)** Changes of the percentages of IFN-γ-secreting CD4^+^ T cells and CD8^+^ T cells pre-therapy and post-therapy in the TB group; **(D)** Changes of the percentages of TNF-a-secreting CD4^+^ T cells and CD8^+^ T cells, and TNF-a-secreting CD14^+^ monocytes pre-therapy and post-therapy in the TB group. *, p<0.05 and **, p<0.01. HT, HIV/TB co-infection.

The changes of cytokine-secreting cells after anti-TB treatment were evaluated in the HT and TB group (shown in **Figure 5**). After one week’s anti-TB treatment, the percentage of CD8^+^ T cells secreting TNF-α was significantly increased in the HT group (p=0.029), the percentage of CD4^+^ T cells secreting TNF-α was also significantly increased in the TB group (p=0.003). All those indicate that TNF-α-secreting cells may respond to the early stage of anti-TB treatment in both HIV/TB co-infection and TB. No apparent changes of the percentages of other proinflammatory, regulatory and cytotoxic cytokine-secreting cells were observed in the HT group after anti-TB treatment (data not shown).

## Discussion

The function and absolute numbers of peripheral cytokine-secreting cells in the natural infection state may suggest different diseases status in particular scenario. To realize precise profile of peripheral cytokine-secreting cells among patients with HIV/TB co-infection in natural infection status, we have investigated the absolute numbers and percentages of pro-inflammatory cytokine-secreting T cell populations and monocytes, as well as the absolute numbers and percentages of regulatory and cytotoxic cytokine-secreting T cells in HIV/TB co-infection by comparing with mono-TB and mono-HIV infection under no MTB-specific stimulation. We found that although the absolute numbers of pro-inflammatory, regulatory and cytotoxic cytokine-secreting cells were significantly lower in the HT group, the function of some of these cells in the HT group were comparable to or even higher than that in the TB and/or HIV groups.

IFN-γ is universally considered to be predominant in Th1 related immunity and plays a central role in protecting against TB and some viruses’ infection (Bustamante et al., 2014). Increased IFN-γ level in PBMCs was found in TB, supporting the widely clinical use of interferon-gamma release assay (IGRA) in determining MTB infection (Gong and Wu, 2021). Previous study also found an enhanced IFN-γ production in CD8^+^ T cells from MTB-infected mice, which might be related to the glycolytic reprogramming (Russell et al., 2019). However, all these researches were based on the detection of MTB-specific IFN-γ production. Our study found that in the natural infection status without MTB-specific stimulation, the IFN-γ production in CD8^+^ T cells in the TB group was still enhanced, but the production of IFN-γ in both CD4^+^ and CD8^+^ T cells in the HT group were not increased. Those were consistent with the evidence that the severe immune deficiency caused by HIV/TB co-infection would influence the production of IFN-γ, and further, influence the accuracy as well as lead to the false negative result of IGRA (Chen et al., 2022). Previous study found that in severe TB, IFN-γ production in PBMCs was reduced, especially in HIV-positive patients (Sodhi et al., 1997). We further observed significantly lower secretion of IFN-γ in CD8^+^ T cells but no significant change of IFN-γ secretion in CD4^+^ T cells in the HT group in comparison to the TB group, suggesting that HIV/TB co-infection might mainly affect the secretion of IFN-γ in CD8^+^ T cells.

TNF-α, mainly produced by monocytes and macrophages, plays an essential role in the formation and maintenance of granulomas in MTB infection (Chen et al., 2014). Elevated MTB-specific TNF-α secretion in PBMCs by ELISA and elevated MTB-specific TNF-α secretion in CD4^+^ and CD8^+^ T cells by ICS have been reported in TB than that in HC (Wang et al., 2013; Lichtner et al., 2015). Previous study on the comparison of children with pulmonary TB and healthy controls by ICS without MTB-specific stimulation found no difference of peripheral TNF-α secretion in CD14^+^ monocytes between them (Torun et al., 2014). However, our study showed a significantly decreased TNF-α production in CD4^+^, CD8^+^ T cells and CD14^+^ monocytes in TB, and HIV co-infected with TB had obviously decreased TNF-α production in CD4^+^, CD8^+^ T cells and CD14^+^ monocytes under a natural infection status without external antigen stimulation. Our results suggested that chronic TB might suppress the TNF-α expression or deplete TNF-α-secretion cells, or TNF-α^+^ cells might be immigrating to the lesion location, which led to the low level of TNF-α^+^ cells in the peripheral blood of TB and HIV/TB co-infection.

In our study, the TB group significantly increased the IL-17A expression in CD8^+^ T cells than the HC group, and the HT group significantly increased the IL-17A expression in CD8^+^ T cells than the HIV group, suggesting that TB might promote IL-17A production in CD8^+^ T cells. IL-17 was known to recruit neutrophils, macrophages, Th1 cells and IFN-γ-producing cells, and accelerate Th1 memory response (Li et al., 2018). Previous study showed that IL-17-secreting CD8^+^ T cells played a role in immunity and protection against TB, and an expansion of MTB antigen-specific IL-17A-producting CD8^+^ T cells was observed in the peripheral blood of patients with tuberculous lymphadenitis (Kumar et al., 2014). Our study also showed an expansion of circulating IL-17A-secreting CD8^+^ T cells in TB, which suggested that CD8^+^ T cells might play a protective role against MTB infection by promoting IL-17A production. Low percentage of circulating IL-17A-secreting CD8^+^ T cells, accompanied by diminished production of IL-17, have been observed in untreated as well as treated HIV-infection, which might be correlated with high cellular and plasma immune activation levels (Perdomo-Celis et al., 2018). Our study found co-infected with HIV decreased the percentage of circulating IL-17A-secreting CD8^+^ T cells in TB, but this decrease wasn’t significant. How HIV/TB co-infection effects on the IL-17A-secreting CD8^+^ T cells need further investigation.

Regulatory T cells (Treg) were reported to be expanded in patients with TB and might contribute to suppression of Th1-type immune responses by secreting TGF-β (Guyot-Revol et al., 2006). Among patients with chronic HIV infection, HIV antigens could trigger the proliferation of virus-specific Treg, and further suppressed HIV-specific effector CD4^+^ and CD8^+^ T-cell responses (Aandahl et al., 2004; Kinter et al., 2004). In our study, we observed elevated ability of TGF-β secretion in Foxp3^+^CD4^+^ T cells in TB and HIV infection, and HIV/TB co-infection further enhanced the production of TGF-β in Foxp3^+^CD4^+^ T cells when compared with TB. High percentage of circulating TGF-β-producing Tregs have been reported in patients with cutaneous tuberculosis, which might be related to the negative regulation of T-cell immune responses (Saini et al., 2018). Elevated TGF-β production in PBMCs from patients with HIV/TB co-infection than from healthy controls was also observed, and high level of TGF-β in HIV patients might be a reason for defective MTB-specific IL-1β, IL-2 production and activation of latent TB in HIV (Devalraju et al., 2019). Our findings hypothesized that the expansion of TGF-β-secreting CD4^+^ Treg cells in HIV/TB co-infection played an important role in inhibiting T-cell immunity against MTB.

We found both TB and HIV infection elevated the production of granzyme A and perforin in CD8^+^ T cells, and HIV/TB co-infection further enhanced granzyme A production in CD8^+^ T cells. Granzymes produced by CTLs could cause programmed cell death in microbes by inducing reactive oxygen species and destroying microbial antioxidant defenses and disrupting biosynthetic and central metabolism pathways required for their survival (Dotiwala and Lieberman, 2019). In both TB and HIV infection, CD8^+^ cytotoxic T lymphocytes (CTLs) were conductive to prevent MTB infection and HIV replication by secreting granzymes and perforin (Saeidi et al., 2015; Voskoboinik et al., 2015). Moreover, increased percentages of circulating CD4^+^ CTLs with secretion of granzymes and perforin have been reported during HIV infection, which played a host-protective role *via* cytolytic activity against HIV-infected cells (Dillon et al., 2022). Consistent with this, we also found HIV infection elevated the production of perforin in CD4^+^ T cells, and HIV/TB co-infection had more granzyme A secretion in CD4^+^ T cells than HIV infection and TB alone. Interestingly, we observed a significantly differential regulation of perforin and granzyme A production in circulating CD8^+^ T cells in the HT group when compared with the TB group. Previous study also found opposite secretion of granzyme B and perforin in CD8^+^ T cells between TB (with and without HIV infection) and healthy people, and in lined with our result, dramatically decreased perforin-secreting CD8^+^ T cells among HIV/TB co-infection than among TB was reported in this study (Sarkar et al., 2016). We speculated that the secretory and regulatory pathways of granzyme A might be independent of the secretory pathway of perforin, and those pathways might be differently affected in TB and HIV/TB co-infection. Our findings suggested that in HIV/TB co-infection, the enhanced granzyme A production in both CD4^+^ CTLs and CD8^+^ CTLs might be critical for the clearance of viruses and MTB.

In our study, significant changes of IFN-γ production in CD4^+^ and CD8^+^ T cells weren’t observed among both TB and HT group after one week’s anti-TB treatment, while significant changes of TNF-α production in CD4^+^ or CD8^+^ T cells were found, suggesting that the change of TNF-α may be more sensitive than IFN-γ in the early stage of anti-TB treatment. Many cytokines were investigated as the marker of anti-TB therapy efficacy, but significant heterogeneity existed between studies due to different research objects, detection methods and observation time (Clifford et al., 2015b; Clifford et al., 2015a). A previous study found that plasma TNF-α level decreased significantly within one week’s treatment in HIV/TB co-infected patients who respond well to anti-TB treatment (Hsieh et al., 1999), while other studies reported an increase in plasma TNF-α level in patients with severe TB following seven days of anti-TB treatment, which was associated with clinical deterioration (Bekker et al., 1998; Eum et al., 2010). In our study, all patients with TB had good responses to seven days’ anti-TB therapy, and only TNF-α showed significant change after seven days’ treatment. Whether TNF-α can be used as an early marker for evaluating the efficacy of anti-TB treatment still need further investigation.

To our knowledge, our study is the first to investigate the functional status of different cytokine-secreting cells in HIV/TB co-infection under a natural infection state in China, and compared them with HIV infection and TB alone. One of the limitations of our study is that the sample size is relatively small, but this preliminary study also have provided a reference for further exploration in this field. Another limitation is the significant gender differences between different groups, particularly in the HIV group. Considering the sample size and other factors, our study may not fully control for the influence of gender. In our future studies, the influence of gender will be given more attention to gain a more comprehensive understanding of the impact of different factors on the secretion of cytokine by immune cells.

## Conclusions

Despite the obviously decreased absolute numbers of peripheral pro-inflammatory, regulatory and cytotoxic cytokine-secreting cells in HIV/TB co-infection, different functional profile of these cells were observed in the natural course of HIV/TB co-infection when compared them with TB and HIV infection alone. TNF-α-secreting CD8^+^ T cells may be more sensitive for evaluating the early efficacy of anti-TB treatment in patients with HIV/TB co-infection.

## Data availability statement

The original contributions presented in the study are included in the article/supplementary material. Further inquiries can be directed to the corresponding authors.

## Ethics statement

The studies involving human participants were reviewed and approved by the institutional ethics committee of Zhongnan Hospital of Wuhan University (2016009). The patients/participants provided their written informed consent to participate in this study.

## Author contributions

KL, YF and LS conceived and designed this investigation. WG, QZ and SS helped to design the scheme of the investigation and collected the original data. YX, YY, LF and ML performed the experiments. YT, SW, WG and SZ analyzed the data. YT, WG and KL contributed to the writing of the paper. All authors contributed to the article and approved the submitted version.
